# Mechanochemical bottom-up synthesis of phosphorus-linked, heptazine-based carbon nitrides using sodium phosphide

**DOI:** 10.3762/bjoc.18.125

**Published:** 2022-09-12

**Authors:** Blaine G Fiss, Georgia Douglas, Michael Ferguson, Jorge Becerra, Jesus Valdez, Trong-On Do, Tomislav Friščić, Audrey Moores

**Affiliations:** 1 Centre in Green Chemistry and Catalysis, Department of Chemistry, McGill University, 801 Sherbrooke Street West, Montréal, Québec, Canada; 2 Department of Chemical Engineering, Laval University, Québec City, Québec, Canada; 3 Facility for Electron Microscopy Research (FEMR), McGill University, Montréal, Québec, Canada; 4 Department of Materials Engineering, McGill University, 3610 University Street, Montréal, Québec, Canada

**Keywords:** carbon nitride, density functional theory, mechanochemistry, phosphorus, photochemistry

## Abstract

Herein, we present the bottom-up, mechanochemical synthesis of phosphorus-bridged heptazine-based carbon nitrides (g-h-PCN). The structure of these materials was determined through a combination of powder X-ray diffraction (PXRD), X-ray photoelectron spectroscopy (XPS), ^31^P magic angle spinning nuclear magnetic resonance (MAS NMR), density functional theory (DFT) and electron energy loss spectroscopy (EELS). Compared to traditional furnace-based techniques, the presented method utilizes milder conditions, as well as shorter reaction times. Both samples of g-h-PCN directly after milling and aging and after an hour of annealing at 300 °C (g-h-PCN300) show a reduction in photoluminescent recombination, as well as a nearly two-time increase in photocurrent under broad spectrum irradiation, which are appealing properties for photocatalysis.

## Introduction

**Scheme 1:**
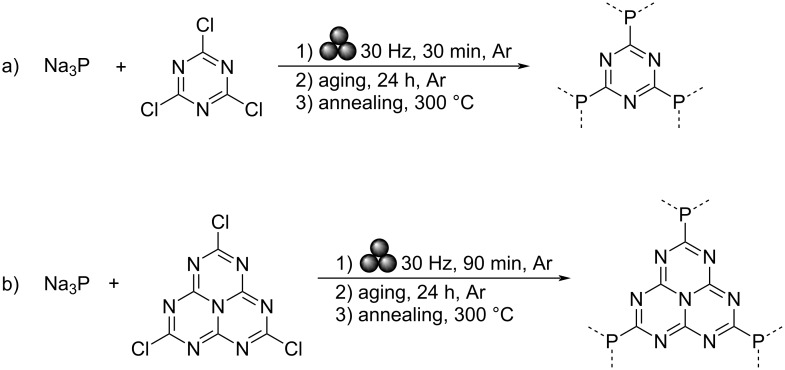
a) Mechanochemical synthesis of g-PCN from sodium phosphide and trichlorotriazine (previous work [38]) and b) g-h-PCN from sodium phosphide and trichloroheptazine (this work).

The development of heteroatom-doped graphitic carbon nitrides (g-CN) has been a rapidly growing area of research since their first report towards water splitting in 2009 [1]. Since that time, the addition of elements such as boron [2], phosphorus [3–5], sulfur and oxygen [6] have shown to help minimize the bandgap of these metal-free photocatalysts, as well as improve their overall stability. Traditional routes to incorporate phosphorus have relied on high-temperature [7] or microwave [8] syntheses, and often proceed through the introduction of a phosphorus atom within the heptazine ring, which constitutes the building block of g-CN, as opposed to in a linking position. Computational studies by Hartley and Martsinovich have investigated the influence of various linkers, including phosphorus atoms, on both the structure and optical behavior of heptazine-based graphitic carbon nitrides [3]. Yet, examples of carbon nitride materials linked together via phosphorus atoms are limited, likely due to challenges in controlling the insertion of phosphorus atoms as linkers under high energy conditions. Mechanochemistry [9–12] has proven to be effective for the synthesis of a variety of polymers [13–17], nanomaterials [18–22], in crystal engineering [23–25], as well as in the synthesis of inorganic materials [26–30] and organic small molecules [31–37]. The ability to avoid bulk solvent and mild reaction conditions allowed by such techniques are beneficial not only from a green chemistry perspective [11], but they also afford conditions conducive to new reactivities and the development of novel materials [9]. Previously, we have explored the synthesis of phosphorus-bridged g-CN-type materials produced from a triazine unit and found that the resulting material featured good photochemical properties (Scheme 1) [38]. Yet, conventional g-CN materials are not based on triazine units, but rather on heptazine ones, thus featuring more open structures. In an effort to replicate a structure closer to known g-CN systems, we explored herein the use of solvent-free, room temperature mechanochemistry to access phosphorus-linked carbon nitride with repeating heptazine units, which were found to show improved photochemistry over pristine graphitic carbon nitride (g-CN). Additionally, the effect of a 1-hour annealing period at 300 °C on the overall structure and photochemical properties of the material was investigated.

## Results and Discussion

Employing a similar method to the one previously developed by our group (Scheme 1a) [38], equimolar amounts of sodium phosphide (Na_3_P) and trichloroheptazine were combined in a vibrational ball mill and milled at 30 Hz for 90 minutes under an argon atmosphere (Scheme 1b). As trichloroheptazine was not readily available commercially, it was synthesized from melem in three steps following a known procedure (see Supporting Information File 1 for the detailed procedure) [4]. The milled powder was then allowed to age under an argon atmosphere for 24 hours, prior to washing via centrifugation in a 3:1 by volume mixture of ethanol and deionized (DI) water. This afforded a material referred to below as g-h-PCN. Alternatively, the sample was annealed for 1 hour at 300 °C under a flow of argon gas, affording the material designated g-h-PCN300.

### Powder X-ray diffraction (PXRD)

**Figure 1:**
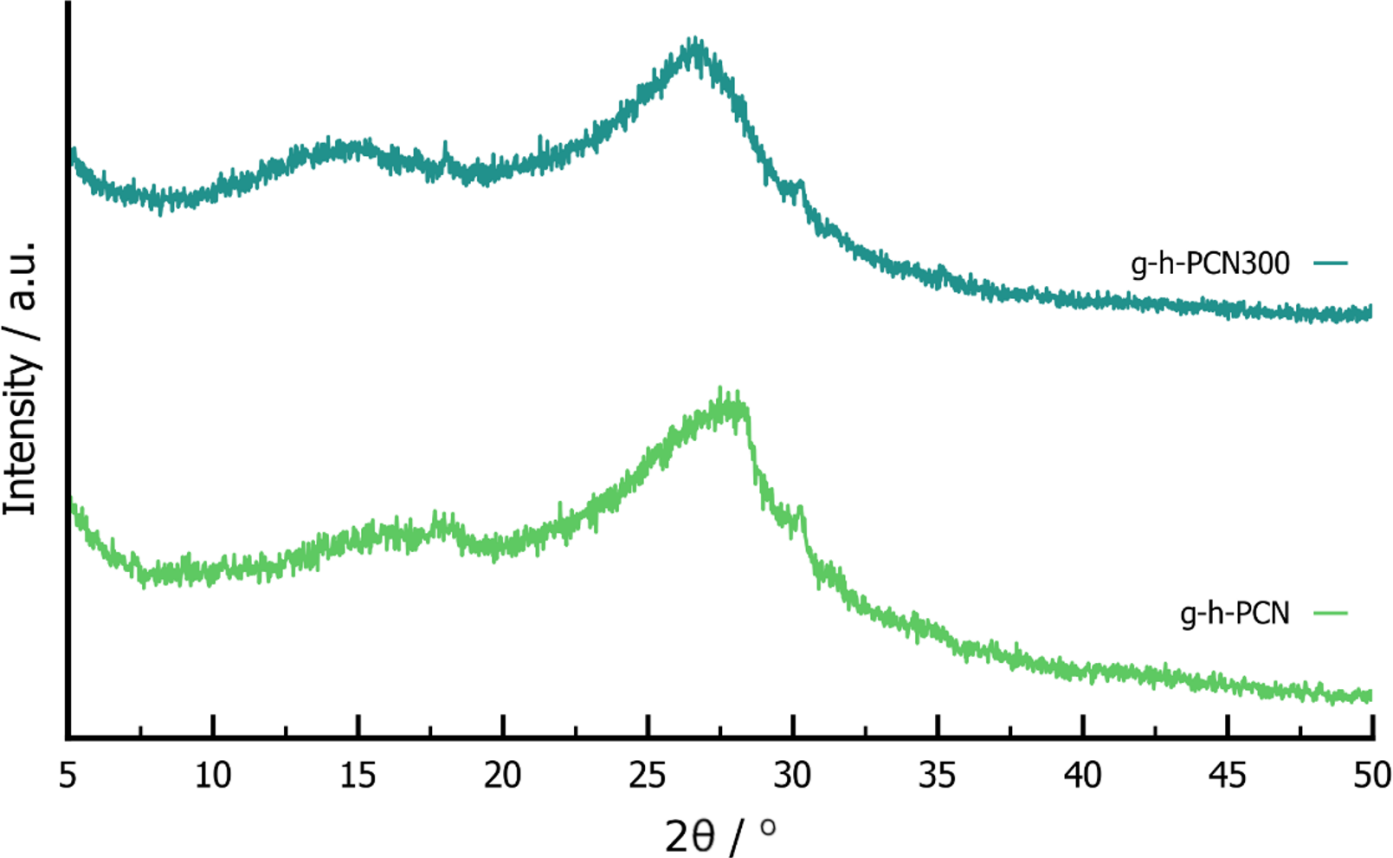
PXRD patterns of g-h-PCN (green) and g-h-PCN300 (teal).

To confirm the formation of a layered structure, powder X-ray diffraction (PXRD) was performed on g-h-PCN and g-h-PCN300 (Figure 1, green and teal). Both g-h-PCN and g-h-PCN300 were largely amorphous but showed two broad Bragg reflections at *2*θ = 16° and 28°. This suggests a high thermal stability of the g-h-PCN structure, being formed during the mild milling and aging conditions, with no need for annealing.

### X-ray photoelectron spectroscopy (XPS)

**Figure 2:**
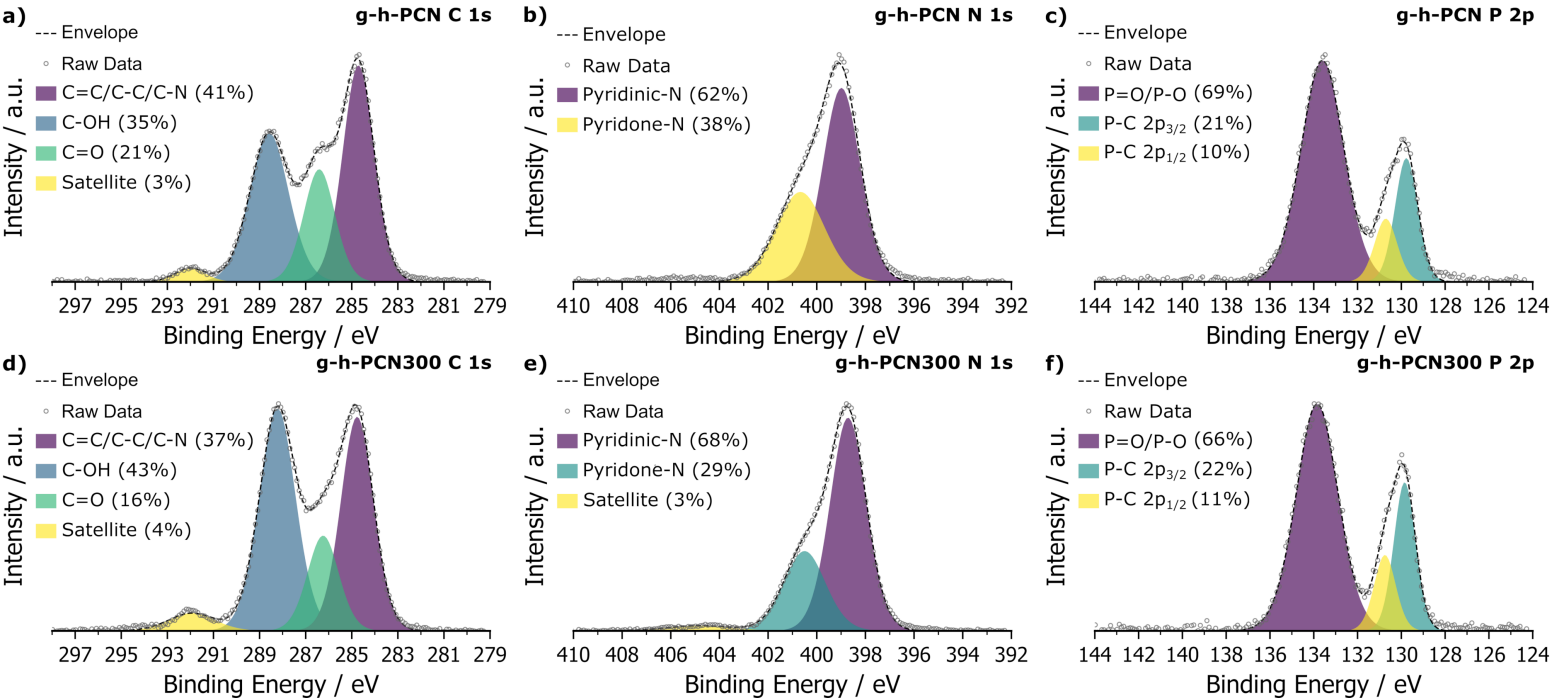
XPS scans of a) C 1s, b) N 1s and c) P 2p for the pre-annealed g-h-PCN and d) C 1s, e) N 1s and f) P 2p focused scans of g-h-PCN300 after annealing at 300 °C for 1 hour.

To gain insight into the atomic speciation within the structure and establish phosphorus atoms are linkers between heptazine units, X-ray photoelectron spectroscopy (XPS) was used to probe the surface. In g-h-PCN, XPS scans focused on carbon 1s showed three major peaks at 284.7, 286.4, and 288.6 eV, corresponding to C=N, C–OH and C=O signals, respectively (Figure 2a), as well as a peak centered on 292.1 eV due to charging effects [39]. The presence of C=N bonds established by XPS indicates that the heptazine structure was preserved during milling and aging with Na_3_P. Nitrogen 1s focused scans of g-h-PCN showed a 62% to 38% ratio of pyridinic to pyridonic-N type nitrogen environments, centered on 399.0 eV and 400.7 eV, respectively [16,40] (Figure 2b). This suggests that while the majority of the heptazine ring remained pyridinic in nature, partial reduction of the ring structure, due to the reductive nature of Na_3_P and mild oxidation during ambient workup following milling and aging, can also have occurred. Additionally, the phosphorus 2p signal in g-h-PCN showed the majority of phosphorus exists as a mixture of P=O and P–O species, with a major peak centered at 133.6 eV (69%, Figure 2c). These species are formed by oxidation with air and hydrolysis upon quenching in water and ethanol at the end of the aging step. g-h-PCN300 featured the same three major carbon 1s peaks at 284.8, 286.2 and 288.2 eV for C=N/C=C, C=O and C–OH species, respectively, as well as the charging peak seen in g-h-PCN. In g-h-PCN300, a reduction of the C=N ratio to 37%, reduction of C=O character from 21% to 18% compared to g-h-PCN and an increase in C–OH character from 35% to 43% suggests mild hydrolysis, likely of terminal trichloroheptazine, during the annealing step at 300 °C, even under a flow of argon gas (Figure 2d). The nitrogen 1s scans show a similar trend, with the ratio of pyridinic and pyridone nitrogen being 68% and 29%, respectively, compared to 62% and 38% in g-h-PCN (Figure 2e). Phosphorus 2p focused scans of g-h-PCN300 showed a slight reduction in P–O and P=O bond character from g-h-PCN to g-h-PCN300 down from 69% to 66% (Figure 2f).

### Analysis by infrared spectroscopy

The structural motifs seen through XPS were further validated by Fourier-transform infrared attenuated total reflectance (FTIR-ATR) spectroscopy. For g-h-PCN, the sharp absorption band at 800 cm^−1^ was indicative of the heptazine breathing mode, typically seen for nitrogen- and phosphorus-linked C_3_N_4_ materials (Supporting Information File 1, Figure S3, purple) [41]. The retention of C=N bonds was also further supported by the observation of a series of bands in the range of 1300–1800 cm^−1^. While the retention of the heptazine ring structure was evident by FTIR-ATR, a low-intensity additional signal at ≈950 cm^−1^ is also seen, indicative of the formation of P–C bonds [42], consistent with the results of XPS analysis (Supporting Information File 1, Figure S3, teal). For the g-h-PCN300 material, the overall spectrum showed similar features to that of g-h-PCN, notably retaining the sharp absorption band corresponding to the heptazine breathing mode at 800 cm^−1^, while also retaining the characteristic P–C vibration at 950 cm^−1^ (Supporting Information File 1, Figure S3, green).

### STEM-EELS analysis

The composition and particle morphology were investigated further using scanning tunneling electron microscopy-electron energy loss spectroscopy (STEM-EELS). The STEM-EELS data for a g-h-PCN sample prior to annealing showed equal distribution of carbon and nitrogen with minimal phosphorus present, and particles roughly 400 nm in length (Supporting Information File 1, Figure S4a). Upon annealing at 300 °C for 1 hour under argon, the phosphorus content is shown to increase, while still remaining partially localized, with the particles retaining their size and morphology (Supporting Information File 1, Figure S4b).

### ^31^P magic angle spinning (MAS) NMR

**Figure 3:**
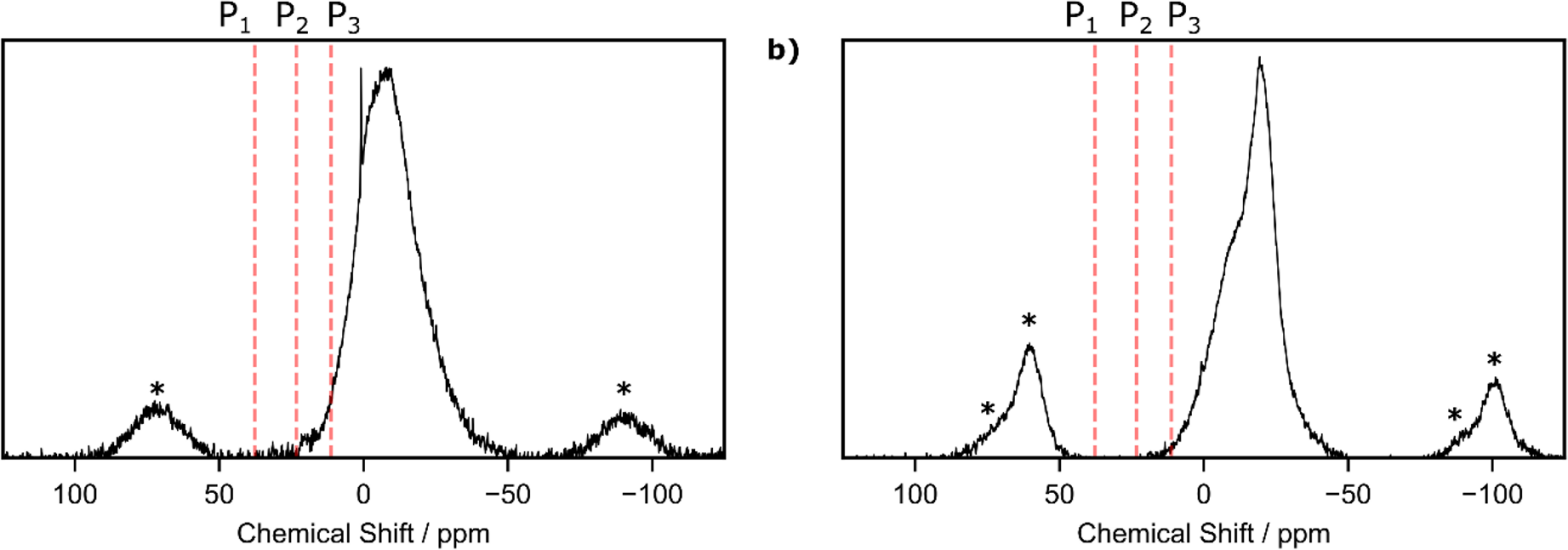
^31^P MAS NMR of a) g-h-PCN and b) g-h-PCN300. Asterisks denote spinning sidebands.

Bulk solid-state analysis of the heptazine-based materials showed similar resonances to previous work by our group on phosphorus-linked triazine networks [38]. The ^31^P MAS NMR of g-h-PCN showed a broad resonance centered around −8.9 ppm, with a sharp residual phosphate resonance at 0.9 ppm (Figure 3a). NMR analysis of similar materials, by our group as well as others, suggest that the broad resonance corresponds to a largely amorphous phase with predominately phosphate and phosphite-like environments, with the broad resonance at −8.9 ppm possibly corresponding to hydrated sodium phosphate byproducts [43–44]. The NMR spectrum of the g-h-PCN300 material showed an up-field shift of all main resonances towards −14.4 ppm and −20.6 ppm (Figure 3b). As previously shown by our group [38], such a shift in main resonance positioning is indicative of the organization of the formed sheets, indicating a layered structure.

### Computational analysis

**Figure 4:**
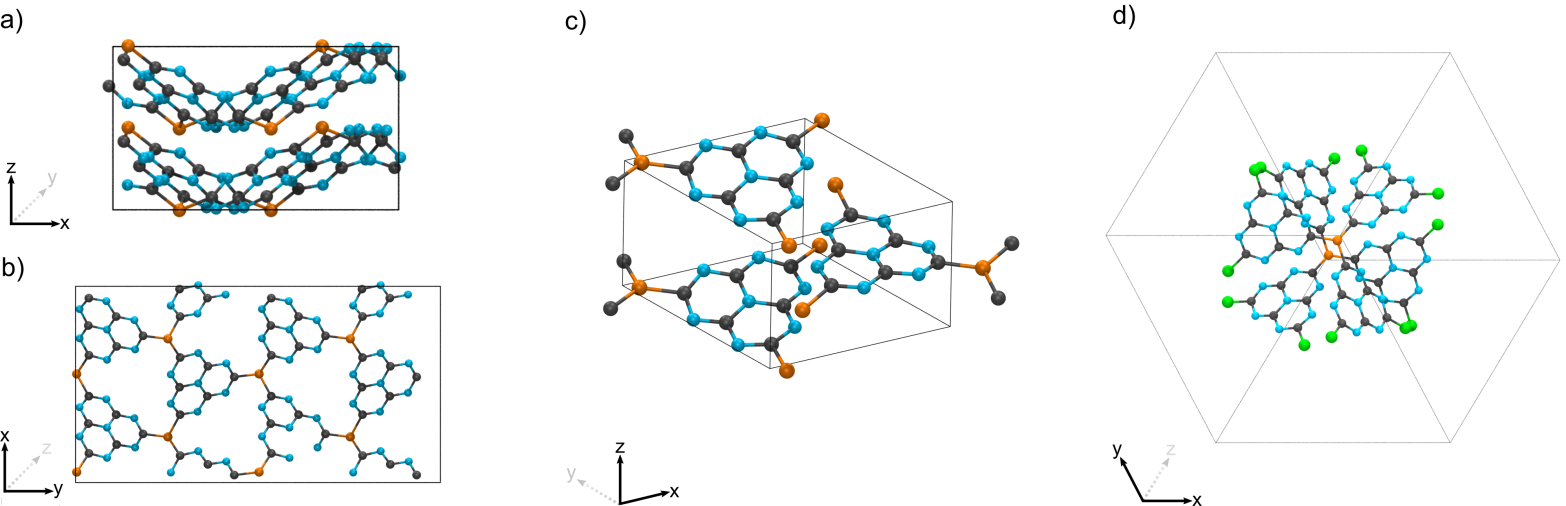
Calculated structures for a) corrugated (edge facing), b) corrugated (single layer), c) layered g-h-PCN and d) phosphine paddlewheel.

The ^31^P NMR chemical shifts were calculated using the plane-wave density function theory (DFT) code CASTEP v20.11 (see Supporting Information File 1 for full computational details) [45]. In the absence of an experimentally resolved crystal structure for g-h-PCN, we followed a similar methodology to our previous work [38] of substituting bridging nitrogen atoms for phosphorus in previously reported heptazine-based graphitic carbon nitrides. We adapted the ab initio predicted structures for a network of corrugated sheets [46] (Figure 4a and 4b) and planar sheets (Figure 4c) [47]. Additionally, we modelled a chlorine terminated monomeric unit based on an experimentally resolved, nitrogen-bridged, paddlewheel structure (Figure 4d) [48]. Calculations resulted in a single chemical environment for the phosphorus atoms in the three model structures. The corrugated and planar network structures have calculated ^31^P chemical shifts at 37.7 ppm (P_1_) and 23.3 ppm (P_2_), respectively, while that of the paddlewheel monomer is at 11.2 ppm (P_3_). The calculated shifts demonstrate phosphorus environments in the mechanochemically synthesized material are distinct to those calculated in ab initio predicted models of the pure reaction product. The variations may be ascribed to the oxygen content found by XPS (Figure 2) or that the mechanochemically synthesized material exists in a different spatial configuration to those previously predicted [46–47].

### Thermal stability

Prior to annealing, g-h-PCN was found to show thermal stability upwards of 200 °C through TGA in nitrogen and air (Supporting Information File 1, Figures S6 and S7). The initial loss seen for both samples is attributed to surface-bound water and carbon dioxide. The g-h-PCN300 retains more mass to upwards of 400 °C in both air and nitrogen, however, at higher temperature (>500 °C), g-h-PCN shows an additional loss of ≈5 wt % compared to g-h-PCN. Overall, under both air and nitrogen, the g-h-PCN retains between 25–35% of its relative mass, up to 800 °C.

### Photochemical activity

The photochemical behavior of both g-h-PCN (Supporting Information File 1, Figure S5a, blue trace) and g-h-PCN300 (Figure S5a, green) was investigated by diffuse reflectance spectroscopy (DRS), and compared to that of a pure g-CN sample made by annealing melamine in a loosely capped alumina crucible at 550 °C for 4 hours, with a ramp rate of 5 °C min^−1^ [49]. The g-CN material was found to exhibit an absorption edge at ≈425 nm (Figure S5a, purple), typical for polymerized and graphitic heptazine materials [50–51]. Both phosphorus-containing structures featured broadened absorption ranges compared to g-CN, with g-h-PCN showing a red-shifted maximum at ≈572 nm (Figure S5a, blue) and g-h-PCN300 (Figure S5a, teal) showing a similar, also red-shifted maximum at 525 nm. Additionally, photoluminescence (PL) measurements showed an initially reduced absorption intensity for g-h-PCN (Figure S5b, blue) compared to that of g-CN (Figure S5b, purple) with further reduction notable in absorption for g-h-PCN300 (Figure S5b, teal). This reduction in photoluminescence has previously been reported for phosphorus-doped carbons and carbon nitrides [52], as the addition of Lewis basic heteroatoms improves the stability of excitons, slowing the rate of recombination. Time-resolved lifetimes showed a marked increase upon replacement of the nitrogen linker for phosphorus. Nitrogen-linked g-CN showed exciton lifetimes of 4.2 µs, while the introduction of a phosphorus linker in g-h-PCN increases the lifetime to 67 µs, with g-h-PCN300 showing lifetimes of 42 µs. We have also observed a similar effect in triazine-based phosphorus-linked graphitic CN structures, with lifetimes of 4.7 and 39 µs seen for g-PCN and g-PCN300, respectively [38].

Improved charge transfer was further confirmed through photocurrent and Nyquist measurements, comparing to pristine g-CN. Photocurrent measurements showed an initial decrease from 10 to ≈5 µA for g-h-PCN (Supporting Information File 1, Figure S4c, blue) compared to pristine g-CN (Figure S4c, purple) respectively after 250 s. The g-h-PCN300 material showed increased photocurrent behavior to both g-CN and g-h-PCN, with photocurrent values of ≈16 µA (Figure S4c, teal), further demonstrating the benefit of both the presence of phosphorus linkages, as well as thermal annealing for the photoactivity. Finally, Nyquist plots (see Supporting Information File 1, Table S1 for details) of g-h-PCN (Figure S4d, blue) and g-h-PCN300 (Figure S4d, teal) showed lower resistivity compared to pure g-CN (Figure S4d, purple), further supporting the idea that the g-h-PCN series enables better charge mobility due to phosphorus linkages.

## Conclusion

Mechanochemistry provided modular, room temperature access to phosphorus-linked carbon nitrides based on heptazine units, through the combination of sodium phosphide and trichloroheptazine. A combination of experimental PXRD, XPS, MAS NMR, as well as theoretical (DFT) approaches confirmed the formation of P–C linkages between repeating heptazine units in the mechanochemically prepared material, with the retention of the heptazine subunits. The introduction of phosphorus linkages reduced photoluminescent recombination and improved exciton lifetimes, when compared to nitrogen-linked g-CN. Overall, this supports future investigations of the room-temperature mechanochemical synthesis of heteroatom containing carbons as well as the benefit of pairing DFT calculations to experimental, structural studies.

## Supporting Information

File 1 General methods and materials as well as additional spectra.
