# Supplementary material for: Mechanochemical bottom-up synthesis of phosphorus-linked, heptazine-based carbon nitrides using sodium phosphide
Source: Beilstein J Org Chem. 2022 Sep 12;18:1203–9. doi: 10.3762/bjoc.18.125 (PMC9490069; doi:10.3762/bjoc.18.125)
Supplement: File 1 — General methods and materials as well as additional spectra. [file Beilstein_J_Org_Chem-18-1203-s001.pdf]

BEILSTEIN JOURNAL OF ORGANIC CHEMISTRY

Supporting Information

for

Mechanochemical bottom-up synthesis of phosphorus-linked, heptazine-based carbon nitrides using sodium phosphide

Blaine G. Fiss, Georgia Douglas, Michael Ferguson, Jorge Becerra, Jesus Valdez, Trong-On Do, Tomislav Friščić and Audrey Moores

Beilstein J. Org. Chem. **2022,** *18,* 1203–1209. doi:10.3762/bjoc.18.125

General methods and materials as well as additional spectra

License and Terms: This is a supporting information file under the terms of the Creative Commons Attribution License (https://creativecommons.org/licenses/by/4.0). Please note that the reuse, redistribution and reproduction in particular requires that the author(s) and source are credited and that individual graphics may be subject to special legal provisions.
The license is subject to the *Beilstein Journal of Organic Chemistry* terms and conditions: (https://www.beilstein-journals.org/bjoc/terms)

1. General methods and materials

Caution: Sodium phosphide readily decomposes in the presence of moist air, and protic organic solvents to produce toxic and pyrophoric phosphane gas, all workups should be conducted in a glovebox or well ventilated fumehood.

All manipulations were conducted in an argon-filled glovebox with O_2 and H_2O levels both at and below 0.5 ppm. Dimethoxyethane (Millipore-Sigma) was dried and distilled under argon over sodium metal with a benzophenone radical indicator. All other solvents were used as received. Sodium phosphide was made using a previously reported method [1] from red phosphorous (Millipore-Sigma), sodium metal (ThermoFischer) and naphthalene (Millipore-Sigma) using standard Schlenk techniques and an argon-filled glovebox. All nanomaterial workups can be conducted under normal, ambient conditions, unless otherwise stated. All chemicals were used as received unless otherwise stated.

Melem (**1**), potassium cyanmelurate (**2**), and 1,5,9-trichloroheptazine (**3**) were synthesized using methods previously reported in the literature [2]. To briefly summarize, melem (**1**) was synthesized by thermal treatment of melamine (Millipore-Sigma) in a covered alumina crucible at 550 °C for 4 h at a ramp rate of 5 °C/min.

<u>Potassium cyanmelurate (**2**)</u>

Melem (3.4 g, 15.8 mmol) was then refluxed in 35 mL of 2.5 M aqueous solution of potassium hydroxide at 110 °C for 4 h, hot filtered to remove any insoluble material, cooled on ice to 0 °C before precipitating the product with 95% ethanol (5.3 g, 15.8 mmol).

<u>1,5,9-Trichloroheptazine (**3**)</u>

Potassium cyanmelurate (5.3 g, 15.8 mmol) was ground together with phosphorus pentachloride (9.7 g, 46.9 mmol) in a mortar and pestle for ca. 30 s until homogeneous.

This powder was added to a 100 mL Schlenk tube using a powder funnel, while under a positive flow of argon. The Schlenk tube was sealed with a glass stopper and heated in an oil bath at 130 °C for 10 h, lifting the stopper every hour under a strong active flow of argon to release overpressure. The resulting pale-yellow powder was washed *via* Soxhlet extraction in dry toluene for 24 h and the toluene fraction was filtered through cotton before drying on a RotoVap. The pale-yellow trichloroheptazine (**3**) (2.6 g, 9.4 mmol) was stored protected from light in an argon-filled glovebox until use.

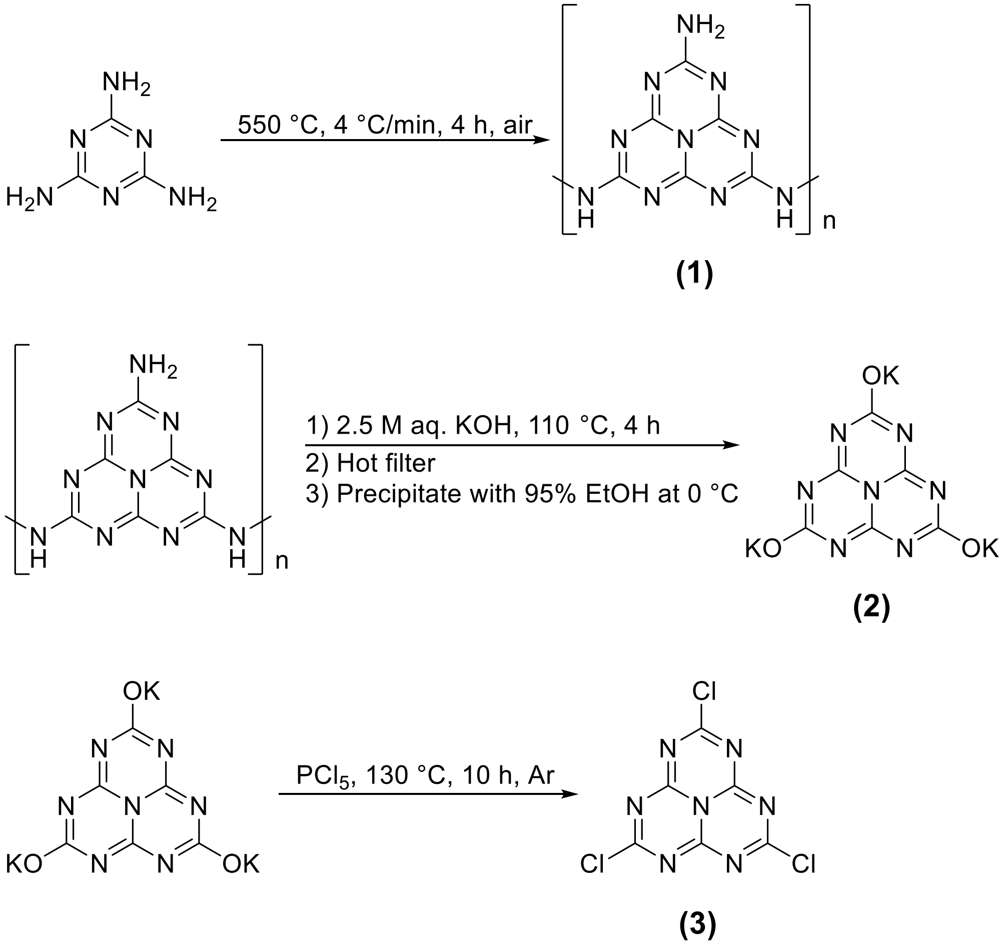

Figure S1: Synthesis of 1,5,9-tri-chloroheptazine from melamine.

1.1. Mechanochemical synthesis of g-h-PCN and g-h-PCN300

To a 10 mL FormTech Scientific zirconia milling jar 1,5,9-trichloroheptazine (0.280 g, 1 mmol) and sodium phosphide (0.1 g, 1 mmol) were added with two 7 mm zirconia balls inside a glovebox. The jar was then sealed with electrical tape, both over the seam and along the length of the jar before taking them out of the glovebox and milling on a Retsch MM400 mixer mill for 90 minutes at 30 Hz. The powder was then washed in 3:1 95% ethanol/water by centrifuging at 10 000 rpm for 15 min. The supernatant was decanted and replaced with 10 mL of water and 30 mL of ethanol, for a total of 3 washings before suspending the g-PCN drying in vacuo at 50 °C for 8 h. Subsequent samples were also annealed by placing in a tube furnace in an alumina boat wrapped loosely with aluminium foil. The boat and atmosphere were purged with a flow of argon for 1 hour before ramping to 300 °C at a ramp rate of 5 °C min^{-1}, holding for an hour at 300 °C and subsequently allowing the sample to naturally cool to room temperature, under a flow of argon.

1.2. Furnace synthesis of P@C_3N_4 reference material

The solid-state synthesis of phosphorus-doped graphitic carbon nitride was adapted from literature [3]. To a 250 mL beaker, melamine (5.0 g) was suspended in 100 mL of DI water. After 30 minutes of stirring at room temperature, 85% phosphoric acid solution (2.8 mL) was added using a micropipette. The suspension was then heated to 80 °C to dryness (approximately 6 h) and the resulting colorless solid (8.0 g) was collected and added to an alumina crucible. The crucible was then loosely capped and heated in a box furnace at 560 °C for 4 h with a ramp rate of 5 °C min^{-1}. The resulting pale-yellow powder (3.0 g) was pulverized in a mortar and pestle before storage.

1.3. Powder X-ray diffraction measurements

Powder X-ray diffraction patterns were acquired over a range of 5° to 50° *2*θ with a step size of 0.02° *2*θ and an integration time of 0.5 s using a Bruker D8 Advantage X-ray Diffractometer equipped with a Cu Kα (λ = 1.5418 Å) source, LinxEye detector, and a Ni filter. Measurements were performed using a silicon zero background holder. PXRD patterns were analyzed using X'Pert HighScore Plus.

1.4. X-ray photoelectron spectroscopy measurements

Samples were analyzed on a Fischer Scientific Kα X-ray spectrometer with an excitation source of Al Kα = 1486.6 eV. The binding energies were corrected by referencing the C 1s line to 284.80 eV. A spot size of 400 μm was used, running 5 survey scans at 200 mV for 50 ms residence times, and 10 scans for specific elements, also at residence times of 50 ms. Deconvolution and peak position were determined using Avantage processing software.

1.5. Solution ^{13}C NMR

^{13}C solution NMR was conducted using a Bruker AVIIIHD 500 MHz NMR spectrometer. Solid sample (≈10 mg) was dissolved in 700 μL of DMSO-d_6 (Millipore Sigma). Spectra were analyzed using MestReNova (v14.2.1)

1.6. ^{31}P MAS NMR analysis of g-h-PCN and g-h-PCN300

Direct polarization ^{31}P solid-state NMR was performed using a Varian 400 MHz VNMRS widebore spectrometer operating at a ^{1}H frequency of 399.76 MHz with a 4 mm double-resonance probe spinning at 13 kHz. The ^{31}P excitation pulse was 2.2 μs long and 1H decoupling was performed using SPINAL-64 decoupling at 110 kHz. For all phosphorylated materials, a recycle delay of 150 s was used and 128 transients were acquired of each sample, for a total experiment time of just under 5.5 h. Sample

integration was compared to an external ammonium dihydrogen phosphate standard acquired in one single scan after 2.5 min in the magnet (to ensure full polarization).

1.7. Prediction of NMR spectra through periodic DFT calculations

Periodic density functional theory calculations were performed using the plane wave code, CASTEP (v20.11) [4]. Input files were generated using the cif2cell program [5]. The plane wave kinetic energy cut off was converged at 700 eV, and the core regions of electron density were described by On-The-Fly ultrasoft pseudopotentials from the CASTEP library. Monkhorst–Pack grids [6] with a k-point spacing of 0.07 2π $Å^{-1}$ were used to sample the Brillouin zone. Structural optimizations were performed suing the PBE [7] functional with the Grimme D3 dispersion correction [8]. Input structures for the paddlewheel monomer, a corrugated bulk, and a planar bulk structure of g-h-PCN were created by adapting the N-bridged equivalents published by Tragl et al., [9] Garcia and Kroll, [10] and the phase 1 structure by Wang et al., [11] respectively. All structures were adapted by replacing the bridging nitrogen atoms with phosphorus. Predictions of the ^{31}P MAS NMR shielding tensors were calculated using the gauge-inducing projector augmented waves (GIPAW) method [12,13] available in CASTEP. Again, core regions of electron density were described by pseudopotentials from the CASTEP library. The online tool MagresView v1.6.2 [14] was used to analysis the magnetic shielding data. The isotropic chemical shifts, δ_{iso}, of the materials were obtained using, $\delta_{iso} = \sigma_{ref} - \sigma_{iso}$. Where σ_{iso} is the isotropic magnetic shielding of the molecule studied and σ_{ref} is the shielding of a given reference species. Ammonium dihydrogen phosphate (ADHP) was chosen as the reference species with its experimental peak known to be 0.9 ppm. The magnetic shielding of ADHP was calculated using the outlined calculation strategy with the same

plane wave energy cutoff and k-point spacing. The initial structure of ADHP was obtained from the Cambridge Structural Database, reference code VITPIA [15].

1.7 FTIR-ATR measurement

Samples were analyzed via FTIR using a Fourier Diagform-Infrared Attenuated Total Reflection PerkinElmer UATR Two Spectrometer through a range of 400 to 4000 cm^{-1}, with a resolution of 1 cm^{-1}. OPUS 7.5 was used to process and analyze the resulting data.

1.8 Scanning transmission electron microscopy - electron energy loss spectroscopy (STEM-EELS)

STEM-EELS measurements were performed using a ThermoFisher Talos F200X transmission electron microscope operated at 200 keV, equipped with a high brightness XFEG Schottky source and a Gatan Enfinium ER 970 EELS spectrometer. The core-loss spectra were corrected using the zero-loss peak (ZLP) shift correction from a Dual-EELS measurement. The dwell time for EELS acquisition on every spectrum image was 0.5 s, with a drift correction at every row. Relative thickness was measured from an area of 100 nm × 100 nm

1.9. Photoluminescence measurements

The photoluminescence (PL) emission spectra and time-resolved PL (TRPL) spectra were recorded in a PTI QuantaMaster 500 spectrofluorometer and TimeMaster Lifetime fluorometer (Horiba, France). PL excitation wavelength was fixed at 350 nm with a slit overture of 2.0 nm and the decay was obtained with a LED with a fixed excitation wavelength of 369 nm and an overture of 0.2 nm for 30 s.

1.10. Photoelectrochemical measurements

A standard three-electrode system was used to determine the photo-electrochemical properties and band structure, using Pt wire and Ag/AgCl as counter and reference

electrodes, respectively. The working electrode was prepared on fluorine-doped tin oxide (FTO) glass (2.5 × 2.5 cm), which was cleaned by sonication in acetone for 3 h and then dried. The FTO slide was spin-coated with 5 mL of slurry, which was obtained by the sonication of 20 mg of photocatalyst in ethanol for 3 h. The transient photocurrent response was performed on an Autolab PGSTAT204 electrochemical workstation with a 0.5 M aqueous solution of Na_2SO_4 as the electrolyte. The photocurrent was measured at a bias voltage of 0.8 V under solar light irradiation (150 W xenon lamp) with a 10 s light on-off cycle.

Table S1: Resistivity of g-CN, g-h-PCN and g-h-PCN300 for Nyquist measurements, using a frequency range of 0 to 100 Hz.

Sample	Maximum resistivity (Ω)	Change in resistivity (Ω)	Change in resistivity (%)
g-CN	7451	0	0
g-h-PCN	6432	1019	14
g-h-PCN300	6235	1216	16

1.11. Thermogravimetric analysis (TGA)

The thermal stability of the samples was subsequently tested using a TA instruments Discovery TGA 5500. Approximately 5 mg of each sample was analyzed in nitrogen or air through a temperature range of 30 to 800˚C at a ramp rate of 5 ˚C min^{-1} using platinum pans. The difference in mass was then calculated using TRIOS Evaluation software.

2. Results and Discussion

2.1 ^{13}C NMR spectrum

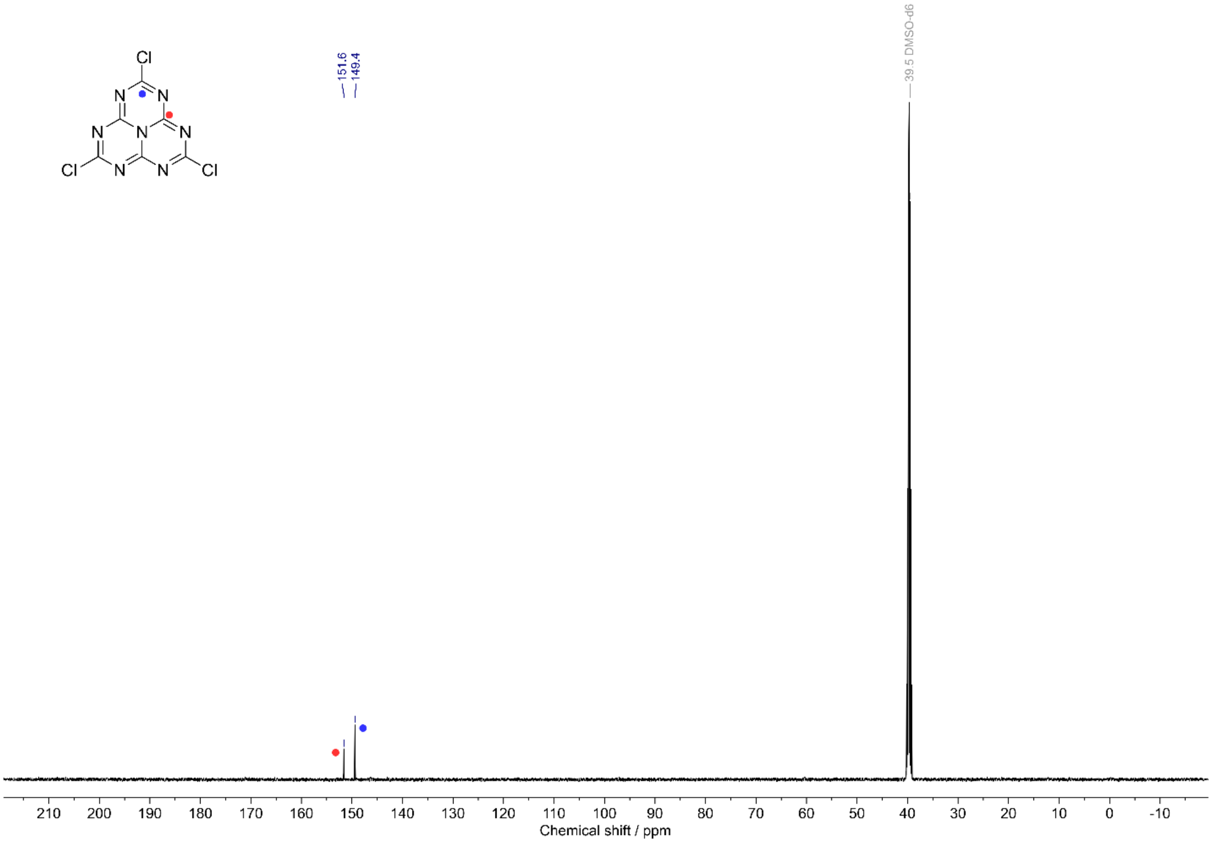

Figure S2: ^{13}C NMR of 1,5,9-trichloroheptazine in DMSO-d_6.

2.2. FTIR-ATR analysis

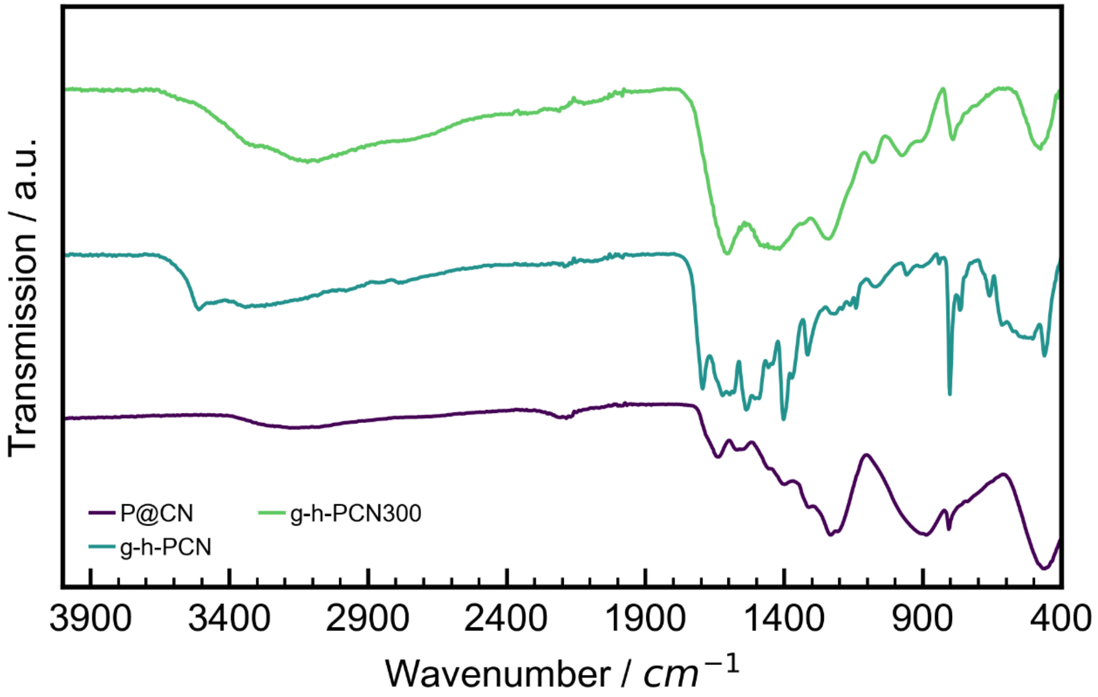

Figure S3: FTIR-ATR of g-h-PCN (green) and g-h-PCN300 (teal).

2.3 STEM-EELS analysis

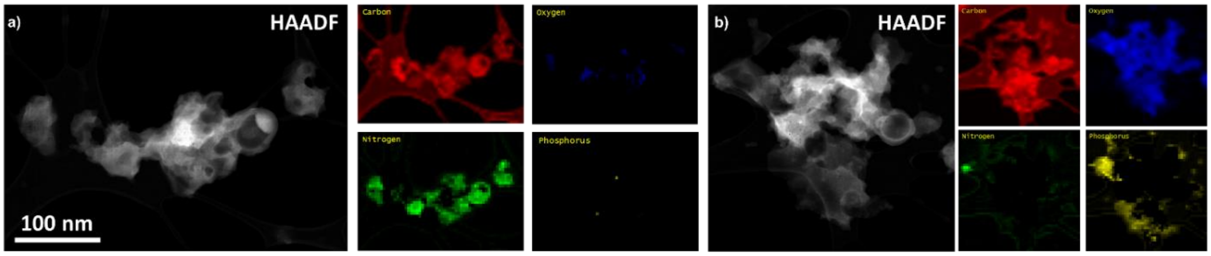

Figure S4: STEM-HAADF images (left) and EELS maps (right) for the characteristic elements presented on graphitic carbon networks. a) g-h-PCN (no annealing) and b) g-h-PCN300 (annealing at 300 °C under argon).

2.4 Photochemical analysis

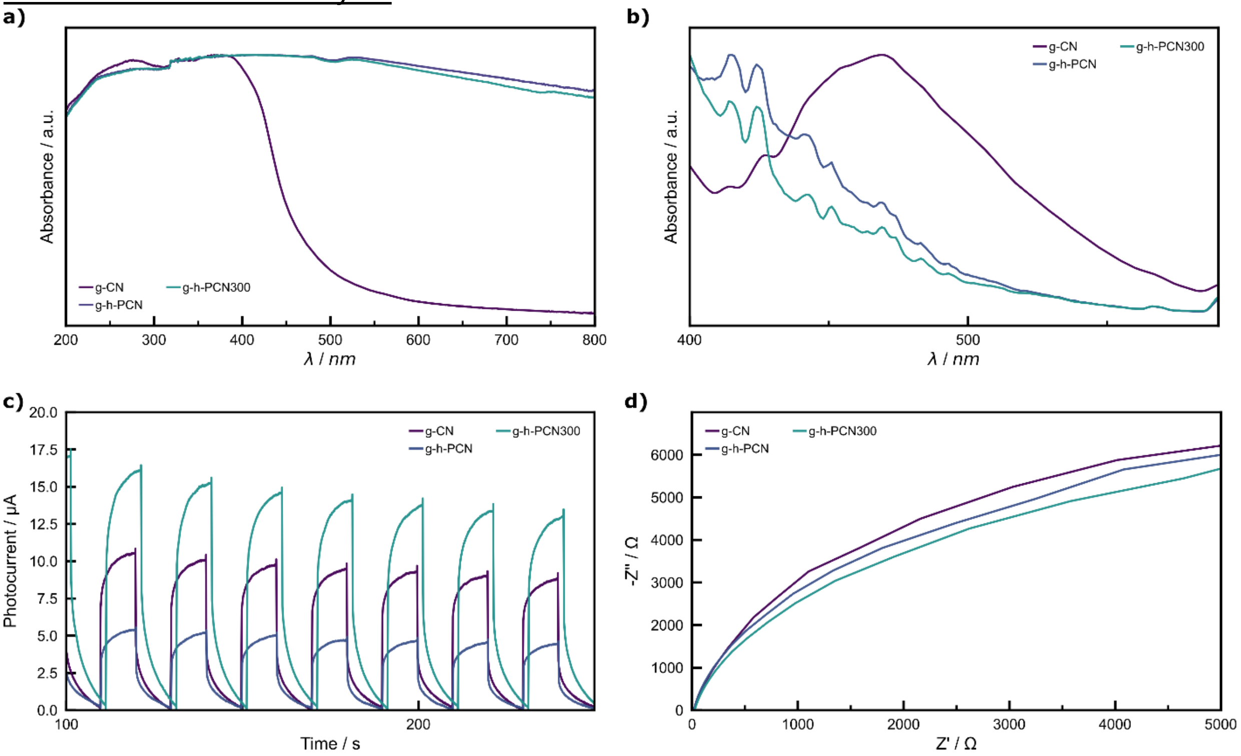

Figure S5: a) Diffuse reflectance spectroscopy b) photoluminescence c) photocurrent and d) Nyquist measurements of g-C_3N_4 (purple), g-h-PCN (blue), , and g-h-PCN300 (green).

2.5 Thermogravimetric analysis

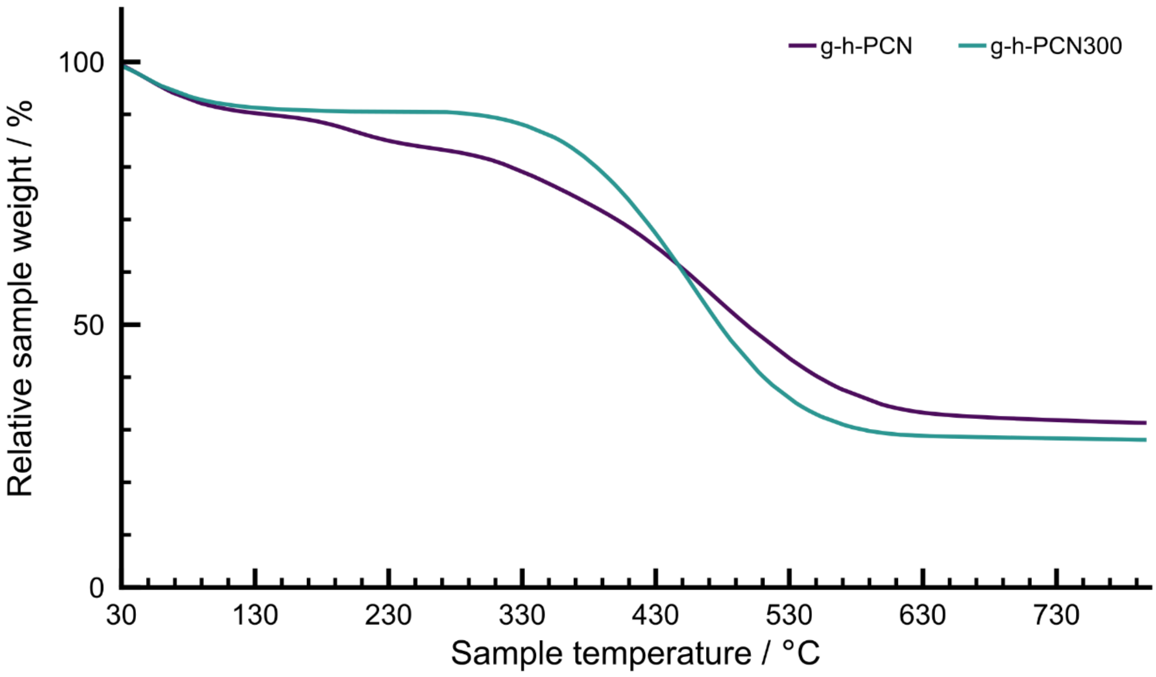

Figure S6: TGA of g-h-PCN (purple) and g-h-PCN300 (teal) from 30 °C to 800 °C under a nitrogen atmosphere, heating at a rate of 5 °C min^{-1}.

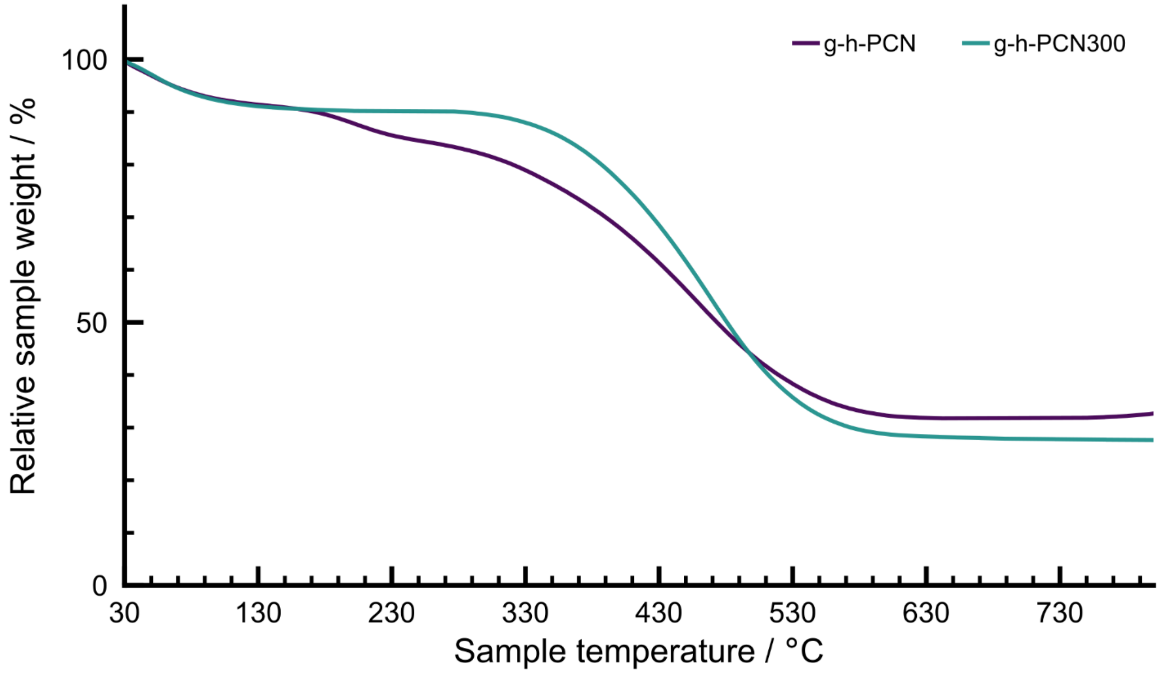

Figure S7: TGA of g-h-PCN (purple) and g-h-PCN300 (teal) from 30 °C to 800 °C under an air atmosphere, heating at a rate of 5 °C min^{-1}.

References

1. Puschmann, F. F.; Stein, D.; Heift, D.; Hendriksen, C.; Gal, Z. A.; Grützmacher, H. F.; Grützmacher, H. *Angew. Chem. Int. Ed.* **2011**, *50*, 8420-3.
2. Kroke, E.; Schwarz, M.; Horath-Bordon, E.; Kroll, P.; Noll, B.; Norman, A. D. *New J. Chem.* **2002**, *26*, 508-512.
3. Ma, J.; Jin, D.; Yang, X.; Sun, S.; Zhou, J.; Sun, R. *Green Chem.* **2021**, *23*, 4150-4160.
4. Clark, S. J.; Segall, M. D.; Pickard, C. J.; Hasnip, P. J.; Probert, M. I. J.; Refson, K.; Payne, M. C. *Z. Kristallogr.* **2005**, *220*, 567-570.
5. Björkman, T. *Comp. Phys. Commun.* **2011**, *182*, 1183-1186.
6. Monkhorst, H. J.; Pack, J. D. *Phys. Rev. B* **1976**, *13*, 5188-5192.
7. Perdew, J. P.; Burke, K.; Ernzerhof, M. *Phys. Rev. Lett.* **1996**, *77*, 3865-3868.
8. Grimme, S.; Antony, J.; Ehrlich, S.; Krieg, H. *J. Chem. Phys.* **2010**, *132*, 154104.
9. Tragl, S.; Gibson, K.; Glaser, J.; Heydenrych, G.; Frenking, G.; Duppel, V.; Simon, A.; Meyer, H.-J. *Z. Anorg. Allg. Chem.* **2008**, *634*, 2754-2760.
10. Gracia, J.; Kroll, P. *J. Mater. Chem.* **2009**, *19*, 3013-3019.
11. Wang, J.; Hao, D.; Ye, J.; Umezawa, N. *Chem. Mater.* **2017**, *29*, 2694-2707.
12. Pickard, C. J.; Mauri, F. *Phys. Rev. B* **2001**, *63*, 245101.
13. Yates, J. R.; Pickard, C. J.; Mauri, F. *Phys. Rev. B* **2007**, *76*, 024401.

14. Sturniolo, S.; Green, T. F. G.; Hanson, R. M.; Zilka, M.; Refson, K.; Hodgkinson, P.; Brown, S. P.; Yates, J. R. *Solid State Nucl. Magn. Reson.* **2016**, *78*, 64-70.
15. Groom, C. R.; Bruno, I. J.; Lightfoot, M. P.; Ward, S. C. *Acta Cryst. B* **2016**, *72*, 171-179.
